# Molecular cytogenetic analysis of a hydatidiform mole with coexistent fetus: a case report

**DOI:** 10.1186/s13256-019-2180-y

**Published:** 2019-08-18

**Authors:** Nozomi Uemura, Yasushi Takai, Yukiko Mikami, Miwa Ogasawara, Masahiro Saitoh, Kazunori Baba, Junichi Tamaru, Masaaki Hara, Hiroyuki Seki

**Affiliations:** 1 Center for Maternal, Fetal and Neonatal Medicine, Saitama Medical Center, Saitama Medical University, 1981 Kamoda, Kawagoe, Saitama 350-8550 Japan; 2 Department of Pathology, Saitama Medical Center, Saitama Medical University, Kawagoe, Saitama 350-8550 Japan; 3 Department of Forensic Medicine, Saitama Medical University, Iruma, Saitama 350-0495 Japan; 4 Sekishindo Hospital, Kawagoe, Saitama 350-1123 Japan

**Keywords:** Complete hydatidiform mole, Partial hydatidiform mole, Postzygotic diploidization, Mole with coexistent fetus

## Abstract

**Background:**

A hydatidiform mole with a coexisting fetus is a rare condition that commonly occurs as either a partial mole with fetus or a twin pregnancy comprising a complete mole and normal fetus. In the former case, the fetus is triploid, and in the latter case, the fetus is diploid with different alleles from those of the mole. Because there is a difference in the persistent trophoblastic disease incidence between the two, an accurate diagnosis is required.

**Case presentation:**

We present a case of a 34-year-old Japanese woman who was pregnant with a hydatidiform mole and two coexisting fetuses. At 17 weeks of gestation, hemorrhage-induced progressive anemia in the mother prompted the decision to terminate the pregnancy, after which no complications occurred. Molecular cytogenetic analysis revealed that one of the fetuses was a normal diploid fetus with the same allele in the fetus and placenta. The hydatidiform mole was revealed to be a mosaic of two diploids, and the other coexisting fetus was a normal diploid that shared one of the mole alleles.

**Conclusions:**

This was presumed to be a rare case of twin pregnancy by triploid embryo formation, followed by loss of an allele due to postzygotic diploidization, development of a diploid fetus, and development of another fetus from a separate embryo. Because of the existence of cases such as this one with a diploid fetus, but without a normal pregnancy coexistent with a complete hydatidiform mole, diagnosis by genetic analysis is required for prognosis.

## Background

Pregnancy in wich the fetus and hydatidiform mole exist simultaneously can be divided into the following two cases. That is, in the case of a twin pregnancy associated with a hydatidiform mole and in the case of a hydatidiform mole with a coexisting fetus, the combined occurrence probability of both is reported as 1 in 22,000 pregnancies [1]. A hydatidiform mole with a coexisting fetus normally exists as a partial mole with a fetus or a twin pregnancy comprising a complete hydatidiform mole and a normal fetus. In the former case, the fetus and mole develop from double sperm fertilization, and cytogenetically, the fetus and mole are often triploid and share the same alleles [2–4]. In the latter case, the complete hydatidiform mole is diploid by androgenesis, and all alleles are from the male parent [5–7]. The coexisting healthy fetus has alleles from both male and female parents and is therefore diploid with different alleles from those of the mole.

We report a case of a hydatidiform mole with a coexisting twin pregnancy with three alleles, where one fetus coexisting with the mole was a normal diploid fetus that shared alleles with the mole and the other fetus with an attached placenta was a normal diploid fetus. To the best of our knowledge, this is the first reported case in which the placenta was a mosaic of two diploids and normal diploid twins coexisted.

## Case presentation

### Patient

**Fig. 1:**
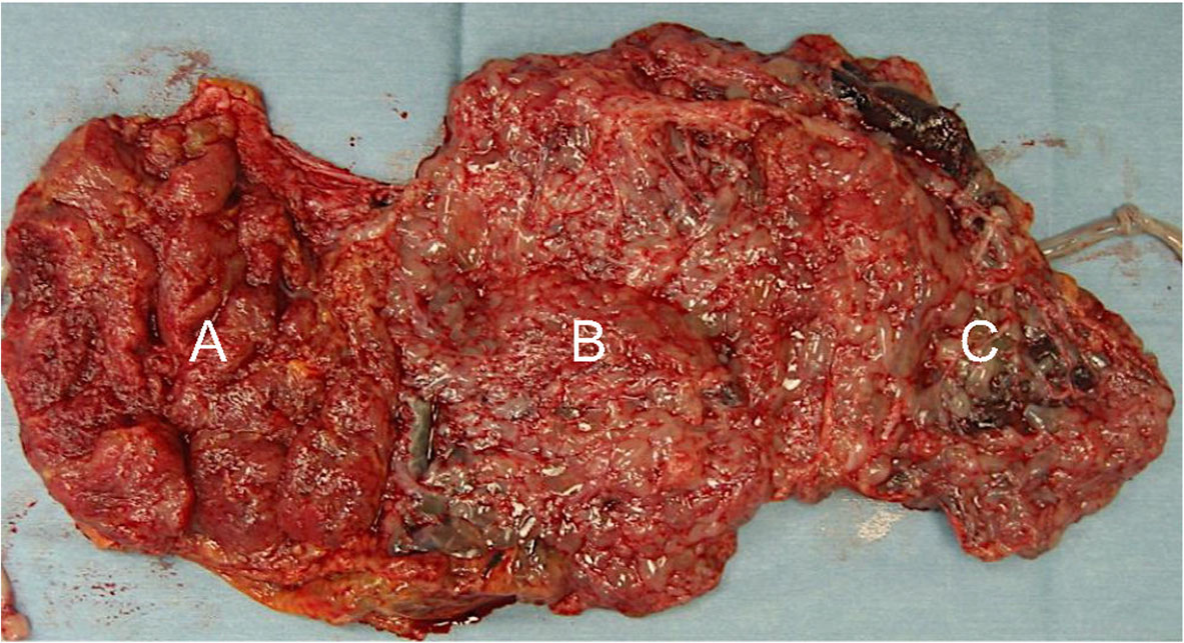
Macroscopic views of fetuses, placentas, and hydatidiform mole. **a** Placenta of fetus 2. **b** Hydatidiform mole. **c** Placenta of fetus 1. The boundary between **b** and **c** was unclear

**Fig. 2:**
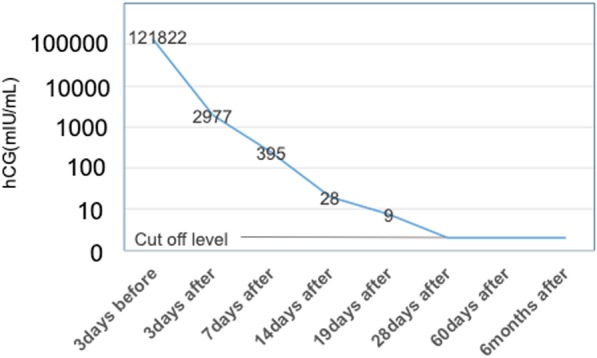
Serum human chorionic gonadotropin (hCG) follow-up before and after termination of pregnancy

A 34-year-old G1P0 Japanese woman with natural conception was determined to have dichorionic diamniotic twins at 6 weeks of gestation and was admitted to a hospital at 13 weeks of gestation for risk of miscarriage. Ultrasound performed at admission revealed molar changes in part of the placenta. At 17 weeks of gestation, the patient was transferred to our hospital for detailed examination. Growth of both fetuses was appropriate for the gestational age, and a coexistent hydatidiform mole was observed. The patient wished strongly to continue the pregnancy, but hemorrhage-induced progressive anemia and growth in the molar tissue were revealed during the next several days, and the patient chose to terminate the pregnancy. The delivered fetuses were both female and had no obvious external malformations. Fetus 1, which was coexistent with the mole, had a slightly yellow skin color that was suggestive of chronic hemorrhage into the amniotic fluid. Macroscopic photographs of the placentas are shown in Fig. 1. The boundary between the normal placenta and hydatidiform mole was unclear. The patient’s serum human chorionic gonadotropin level was followed after termination. It dropped progressively until it became negative after 28 days (Fig. 2). The prognosis of the mother after surgery was good, and she was granted permission for subsequent pregnancies (Table 1).

**Table 1:** Timeline

Gestational age: 6 weeks	Diagnosis of dichorionic diamniotic twins.
Gestational age: 13 weeks	Molar changes were observed in part of placenta.
Gestational age: 17 weeks	The patient was transferred to our hospital. Because of progressive anemia and growth of molar tissue, the patient chose to terminate the pregnancy.
Six months after surgery	The prognosis of the patient after surgery was good. Permission was given for subsequent pregnancies.

### Molecular cytogenetic analysis

**Fig. 3:**
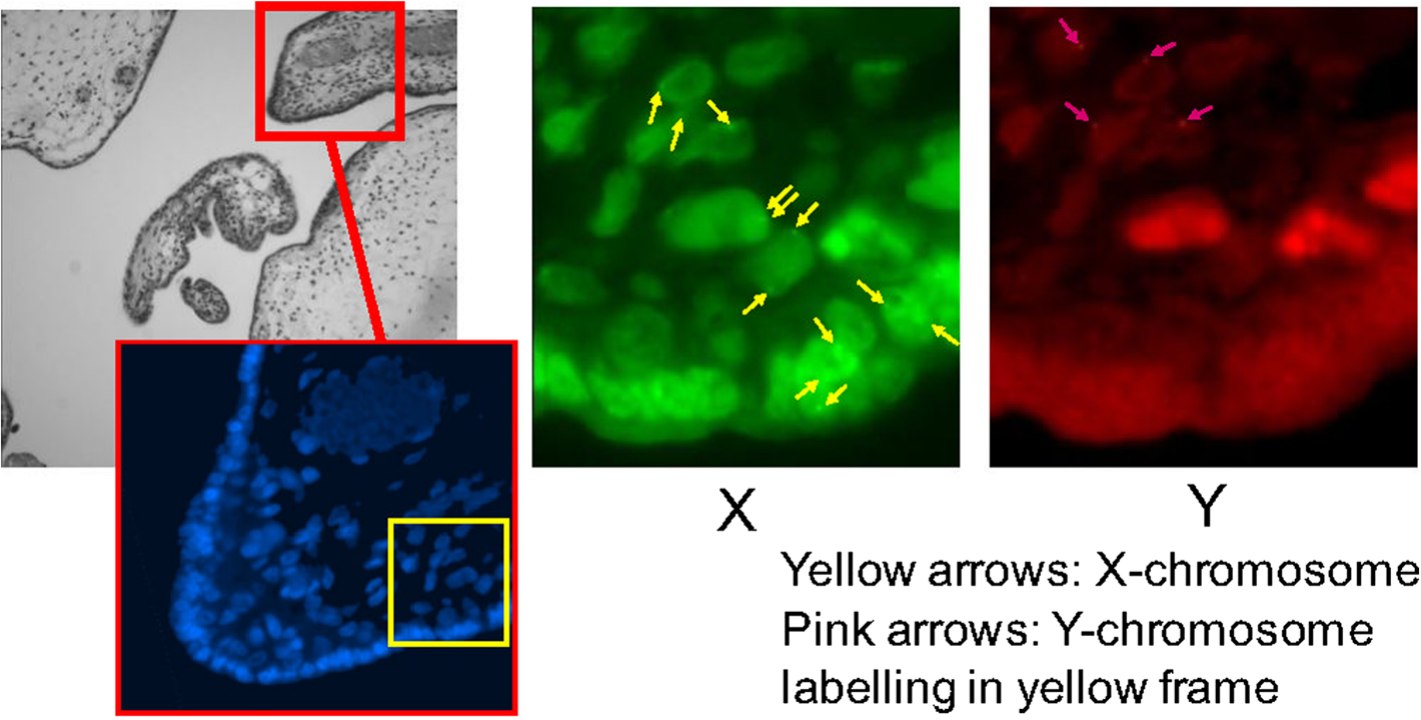
Fluorescence *in situ* hybridization analysis of hydatidiform mole. *Left panel*: Full image of H&E stain and nucleic labeling with 4′,6-diamidino-2-phenylindole in red frame. *Middle panel*: Two X chromosome signals detected in majority of cells. *Right panel*: One X chromosome signal and one Y chromosome signal detected in some cells. Yellow arrows point to a glowing X-chromosome. Pink arrows point to a glowing Y-chromosome

Molecular cytogenetic analysis was performed with written consent from the patient. The patient also granted consent for publication of this report. Short tandem repeat (STR) analysis was performed by SRL, Inc. (Tokyo, Japan), and fluorescence *in situ* hybridization (FISH) analysis was performed by Nihon Gene Research Laboratories, Inc. (Sendai, Japan). As shown in Table 2, two alleles from the male parent and one from the female parent were detected in the mole, and fetus 1, coexistent with the mole, shared all its alleles with the mole. In FISH analysis, the karyotype of fetus 1, coexistent with the mole, was determined to be 46,XX and that of the mole was a mosaic pattern of 46,XY/46,XX (Fig. 3). Meanwhile, fetus 2 with a normal placenta was a normal diploid fetus with one allele from the female parent and one from the male parent.

**Table 2:** Short tandem repeat analysis of peripheral blood lymphocytes from both parents, umbilical cord of fetus 1, and hydatidiform mole

	AMEL	D8S1179	D21S11	D18S51	D19S433	TH01
Mother	X	10, 13	31, 31.2	12, 14	14	6, 9
Father	X, Y	15	30, 32.2	15, 17	13, 14	9
Fetus 1	X	10, 15	30. 31	12, 17	14	6, 9
HM	X, Y	10 15	30, 31, 32.2	12, 15, 17	14	6, 9
	FGA	D3S1358	vWA	D16S539	D2S1338	
Mother	21, 24	15, 18	14, 16	9, 10	22, 25	
Father	22, 23	16	16, 17	12	23	
Fetus 1	21, 22	16, 18	14, 16	9, 12	22, 23	
HM	21, 22, 23	16, 18	14, 16, 17	9, 12	22, 23	

*AMEL* Amelogenin, *HM* Hydatidiform mole

D8S1179, D21S11, D18S51, D19S433, THO1, FGA, D3S1358, vWA, D16S539, D2S1338: 10 short tandem repeat loci present in the human genome

## Discussion

We initially expected this to be a case of twin pregnancy with a normal fetus and partial hydatidiform mole, or a case of trizygotic triplets comprising two normal twin fetuses and a complete hydatidiform mole. However, contrary to our expectation, STR analysis revealed the mole to have three alleles, with two from the male parent and one from the female parent. Meanwhile, FISH analysis revealed diploid cells and showed similarity between alleles of fetus 1 and the hydatidiform mole. Both fetuses were also normal diploid fetuses. After considering the mechanism by which this case arose, the involvement of postzygotic diploidization was suspected. A diploid complete hydatidiform mole is normally a diploid comprising a paternally derived genome created when a sperm fertilizes an empty egg. However, a recent report [8] examined 162 cases of diploid hydatidiform moles and found genomes from both parents in 11 cases. In 3 of 11, they identified one biparental cell population; however, in 8 of 11, they found mosaicism, with one biparental cell population and one androgenetic cell population. The authors identified various possible postzygotic abnormalities, including postzygotic diploidization.

**Fig. 4:**
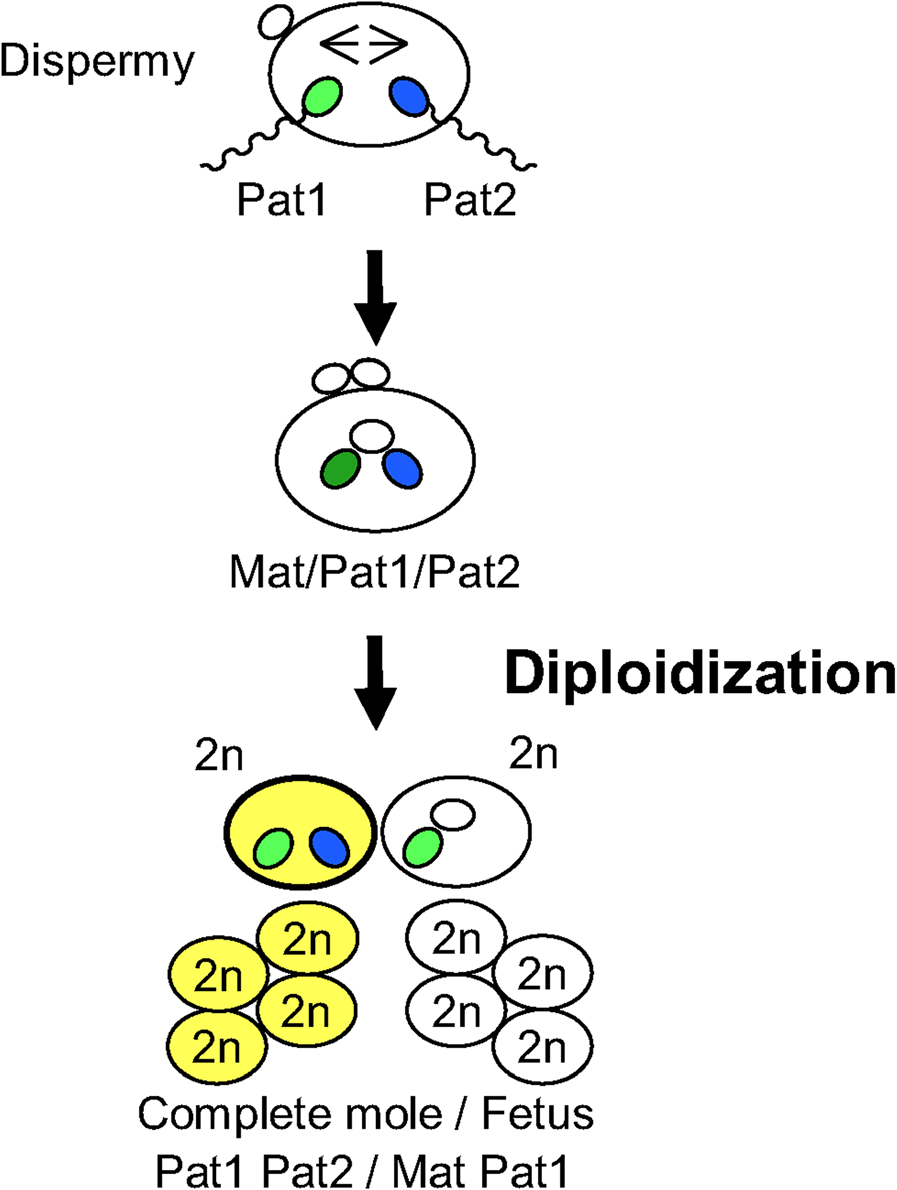
The speculated pathogenic mechanism in the present case. For one of the twins, a triploid embryo formed by dispermy, followed by diploidization of the embryo by somatic cell division; then, a normal placenta and fetus developed from cells with Mat+Pat1, and a hydatidiform mole developed from cells with Pat1 + Pat2

In the present case, one or both of the father’s alleles, but only one of the mother’s alleles, was detected in the hydatidiform tissue, and the tissue included the complete fetus 1 haplotype. Considering this, we presumed a mechanism whereby, for one of the dizygotic twins, a triploid embryo formed by double sperm fertilization. Thereafter, one allele was lost by postzygotic diploidization. Following this, a hydatidiform mole arose with a mosaic karyotype consisting of diploid cells with only a paternally derived genome and diploid cells with a genome from both parents, and a normal diploid fetus and normal placenta also developed with a genome from both parents (Fig. 4).

The possibility of a hydatidiform mole and coexisting fetus developing from diploidization of a triploid embryo has been described, but the development is extremely rare. Past reports have described the occurrence of a partial hydatidiform mole and coexisting diploid fetus developing from postzygotic diploidization, but the hydatidiform mole was not diagnosed or the fetus disappeared within 1 week of fertilization [9, 10]. Ours is considered a rare case in which, after the triploid embryo developed, postzygotic diploidization caused the loss of one allele, after which a diploid fetus developed.

The majority of cases of a mole coexisting with a fetus are normally accounted for by a partial hydatidiform mole or twin pregnancy of a complete hydatidiform mole and a normal fetus. The former case almost always leads to intrauterine death by the second trimester. The latter case can lead to a live birth, but the possibility of persistent trophoblastic disease (PTD) is reported to be relatively high at 53–55% [11]. On this basis, when there is a very strong desire for childbirth, it is recommended that the decision to continue pregnancy be made with fully informed consent after performing chromosome analysis of the fetus by amniocentesis and confirming diploidy. However, as observed in the present case, there are also cases of a diploid fetus that do not involve a normal fetus coexistent with a complete hydatidiform mole. Such cases are also expected to have a different frequency of an increased risk of PTD. Although an accurate frequency of PTD is unknown, considering the absence of PTD in the present case, the frequency is probably lower than that in cases with a complete hydatidiform mole.

## Conclusions

This was a rare case of dizygotic twins developing from a normal embryo, with simultaneous development of a normal karyotype fetus and a mole caused by postzygotic diploidization. In this case, genetic analysis was considered necessary for a definite diagnosis and also for providing accurate information to the patient.

## Data Availability

The authors agree to make the images and data described in the report freely available for use.

## Abbreviations

FISH: Fluorescence *in situ* hybridization
PTD: Persistent trophoblastic disease
STR: Short tandem repeat

## References

[1] Jones WB, Lauersen NH. Hydatidiform mole with coexistent fetus. Am J Obstet Gynecol. 1975;122:267–72. 10.1016/0002-9378(75)90165-9 1130449

[2] Jacobs PA, Szulman AE, Funkhouser J, Matsuura JS, Wilson CC. Human triploidy: relationship between parental origin of the additional haploid complement and development of partial hydatidiform mole. Ann Hum Genet. 1982;46:223–31. 10.1111/j.1469-1809.1982.tb00714.x 7125594

[3] Lawler SD, Fisher RA, Dent J. A prospective genetic study of complete and partial hydatidiform moles. Am J Obstet Gynecol. 1991;164:1270–7. 10.1016/0002-9378(91)90698-q 1674641

[4] Zaragoza MV, Surti U, Redline RW, Millie E, Chakravarti A, Hassold TJ. Parental origin and phenotype of triploidy in spontaneous abortions: predominance of diandry and association with the partial hydatidiform mole. Am J Hum Genet. 2000;66:1807–20. 10.1086/302951 PMC1378061 10801385

[5] Kajii T, Ohama K. Androgenetic origin of hydatidiform mole. Nature. 1977;268:633–4. 10.1038/268633a0 561314

[6] Wake N, Takagi N, Sasaki M. Androgenesis as a cause of hydatidiform mole. J Natl Cancer Inst. 1978;60:51–7. 10.1093/jnci/60.1.51 628023

[7] Yamashita K, Wake N, Araki T, Ichinoe K, Makoto K. Human lymphocyte antigen expression in hydatidiform mole: androgenesis following fertilization by a haploid sperm. Am J Obstet Gynecol. 1979;135:597–600. 10.1016/s0002-9378(16)32983-0 92196

[8] Sunde L, Niemann I, Hansen ES, Hindkjaer J, Degn B, Jensen UB, Bolund L. Mosaics and moles. Eur J Hum Genet. 2011;19:1026–31. 10.1038/ejhg.2011.93 PMC3190256 21654731

[9] Baergen RN, Kelly T, McGinniss MJ, Jones OW, Benirschke K. Complete hydatidiform mole with a coexistent embryo. Hum Pathol. 1996;27:731–4. 10.1016/s0046-8177(96)90406-2 8698320

[10] Golubovsky MD. Postzygotic diploidization of triploids as a source of unusual cases of mosaicism, chimerism and twinning. Hum Reprod. 2003;18:236–42. 10.1093/humrep/deg060 12571155

[11] Bruchim I, Kidron D, Amiel A, Altaras M, Fejgin MD. Complete hydatidiform mole and a coexistent viable fetus: report of two cases and review of the literature. Gynecol Oncol. 2000;77:197–202. 10.1006/gyno.2000.5733 10739712

